# Choroid plexus volume in metabolic syndrome: a cross-sectional neuroimaging study

**DOI:** 10.3389/fneur.2026.1912049

**Published:** 2026-10-16

**Authors:** Xinxin Huo, Tao Chen, Zhenyu Cheng, Boyao Chen, Fushuai Zhang, Yiwen Chen, Pengcheng Liang, Nan Zhang, Yena Che, Changhu Liang, Lingfei Guo, Meng Li

**Affiliations:** 1 Department of Radiology, Shandong Provincial Hospital Affiliated to Shandong First Medical University, Jinan, Shandong, China; 2 Department of Clinical laboratory, Jinan Maternity and Child Care Hospital Affiliated to Shandong First Medical University, Jinan, Shandong, China; 3 School of Medical Imaging, Shandong Medical and Pharmaceutical University, Yantai, Shandong, China; 4 Department of Medical Genetics, Shandong Provincial Hospital Affiliated to Shandong First Medical University, Jinan, Shandong, China; 5 Department of Psychiatry and Psychotherapy, Jena University Hospital, Jena, Germany

**Keywords:** choroid plexus, cognitive function, high-density lipoprotein, magnetic resonance imaging, metabolic syndrome

## Abstract

**Objectives:**

Our investigation aimed to explore the associations between choroid plexus (CP) volume and metabolic syndrome (MetS) components and cognitive-motor performance in a cross-sectional sample.

**Methods:**

A total of 211 participants (73 with MetS and 138 non-MetS controls) were included in this study. CP volume was automatically segmented from 3D T1-weighted images using a 3D nnU-Net. CP volume was normalized to estimated total intracranial volume (eTIV) and expressed as the CP/eTIV ratio. Multivariable linear regression analyses and Spearman correlation analyses were used to examine associations among the CP/eTIV ratio, MetS components and cognitive-motor domains. The Boruta algorithm was applied for exploratory feature selection, and receiver operating characteristic (ROC) analysis was used to evaluate the discriminative capacity of the selected variables for identifying participants with lower global cognitive-motor performance (lowest 25th percentile of global cognitive-motor composite).

**Results:**

Participants with MetS exhibited a higher CP/eTIV ratio than non-MetS controls, and higher CP/eTIV was independently associated with lower high-density lipoprotein cholesterol (HDL-C) levels (*FDR q* = 0.024) after full adjustment and FDR correction. In unadjusted analyses, the CP/eTIV ratio was correlated with multiple cognitive-motor domains. However, these associations were no longer statistically significant after adjustment for confounders. Boruta analysis identified CP/eTIV, white matter hyperintensity (WMH) volume/eTIV, and BMI as key variables, and the combined model yielded an AUC of 0.787 (95% CI: 0.718 to 0.854) for discriminating participants with lower global cognitive-motor composite. DeLong's test showed that the combined model did not significantly outperform CP/eTIV alone (0.742 vs. 0.787; *FDR q* = 0.084) or the model of WMH/eTIV and BMI (0.729 vs. 0.787; *FDR q* = 0.084).

**Conclusion:**

A higher CP/eTIV ratio was associated with MetS and lower HDL-C levels, but was not independently associated with cognitive-motor performance after multivariable adjustment. CP structural variation may represent an imaging correlate of metabolic disturbance, and its relevance to cognitive vulnerability requires prospective validation.

## 1 Introduction

Metabolic syndrome (MetS) comprises abdominal obesity, impaired fasting glucose, hypertriglyceridemia, low high-density lipoprotein cholesterol (HDL-C), and hypertension. This cluster is associated with increased risks of cardiovascular disease and type 2 diabetes mellitus (1). Atherogenic lipid indices, particularly non-HDL-C, are also closely associated with MetS (2). The prevalence of MetS is increasing annually, and more than a billion people suffer from this syndrome worldwide (3). In later stages, MetS is associated with end-organ damage involving the cardiovascular, gastrointestinal, renal, and neurological systems (1, 4). MetS and its components contribute to different aspects of cognitive impairment and are associated with brain aging and neurodegenerative diseases, especially obesity-related indicators (5–7). Magnetic resonance imaging (MRI) has played a significant role in cerebral morphological and functional abnormalities associated with MetS (4). A recent systematic review and meta-analysis further showed that MetS and its components are associated with adverse changes in brain volume, white matter hyperintensity burden, and white matter integrity (8). Variation in glycosylated hemoglobin (HbA1c) has also been associated with structural and functional brain changes, even in normoglycemic populations (9). Metabolic risk factors have been associated with structural brain alterations, including changes in subcortical regions and reduced gray matter volume, and these alterations in brain morphology are strongly linked to the regions with the highest density of endothelial cells and microglia (10, 11). Cerebrovascular dysfunction, endothelial injury, blood–brain barrier (BBB) impairment, and inflammation may link metabolic disease to Tau-related neurodegeneration (12). However, the brain structures that may reflect the interface between peripheral metabolic dysfunction and central inflammation remain incompletely understood.

Located in each ventricle of the brain, the choroid plexus (CP) is a vascularized structure surrounded by epithelial cells. These cells form the blood–CSF barrier through their tight junctions, and the CP is essential for cerebrospinal fluid (CSF) production (13, 14). It not only is involved in the circulation of CSF but also produces a variety of proteins with neuroprotective and developmental properties (15). In addition, after damage to the central nervous system (CNS), CP acts as a site for monocyte recruitment, which assists in repairing the integrity of barriers and reducing brain inflammation (16, 17). This action may be a driving force behind multiple pathological pathways in MetS, leading to abnormalities in brain anatomical structure and cognitive-motor performance (18). Recently, a study indicated that a significantly increased CP volume was observed in patients with mild cognitive impairment (MCI), independent of brain atrophy, suggesting altered CP function in MCI (19). Structural and functional abnormalities of the CP are also involved in the pathophysiological mechanisms of cognitive impairment in neurodegenerative diseases, such as Alzheimer's disease(AD) (20) and Parkinson's disease (21). These findings support a broader relationship between CP abnormalities and cognitive impairment in several neurodegenerative and neuroinflammatory diseases. However, whether this relationship extends to MetS, a systemic metabolic disorder characterized by metabolic dysregulation and chronic low-grade inflammation, remains unclear.

Previous studies have paid more attention to the internal relationship between metabolic disorder risk factors alone and alterations in CP volume (22). Despite MetS being a multifaceted condition that involves dysregulated glucose and lipid metabolism and its correlation with decreased cognitive-motor performance and brain inflammation (7, 18), no clinical trials have explored the underlying pathological mechanisms connecting CP with these phenomena. Therefore, our investigation aimed to explore the associations of CP volume with five components of MetS and multiple neuropsychological tests and to evaluate its ability to discriminate participants with lower global cognitive-motor performance. We propose that (1) there is a significant difference in CP volume between patients with MetS and non-MetS; (2) this alteration in CP volume is affected by MetS status, cumulative component burden, and individual metabolic components; and (3) CP volume is independently associated with multidomain cognitive-motor performance after accounting for demographic and ventricular factors.

## 2 Materials and methods

### 2.1 Subjects

A total of 625 clinical participants were recruited for this prospective study from our hospital between 2021 and 2024. Participant recruitment from 2021 to 2023 was conducted under approval SZRJJ: NO. 2019-002, and continued recruitment and data collection in 2024 were approved under approval SZRJJ: NO. 2024-398. All participants provided written informed consent before enrollment. The cohort was classified into MetS group or non-MetS group according to the Chinese Medical Association Diabetes Branch (CDS) criteria (23) and was diagnosed by experienced endocrinologists. MetS can be diagnosed if any of three or more of the following conditions are met: (a) body mass index (BMI)≥25 kg/m^2^; (b) fasting plasma glucose (FPG)≥6.1 mmol/L or current treatment for diabetes; (c) hypertension: blood pressure≥140/90 mmHg or receiving treatment; or (d) triglycerides (TG)≥1.7 mmol/L; (e) HDL-C < 0.9 mmol/l (men) or < 1.0 mmol/L (women). Participants without MetS were recruited from the same source population and were required not to meet the diagnostic criteria for MetS.

To ensure diagnostic consistency and accuracy, basic patient information was obscured during diagnosis, and inconsistent diagnostic results were excluded. Figure 1A shows a detailed flow chart with inclusion and exclusion criteria. The inclusion criteria were as follows: (1) age > 40 years; (2) complete blood biochemical data; (3) complete neuropsychological assessment; and (4) availability of MRI data of sufficient quality for analysis. The exclusion criteria were as follows: (1) poor-quality MRI scans; (2) structural brain abnormalities on brain MRI (including cerebral small vessel disease (CSVD) burden score ≥ 2); (3) inability to complete the neuropsychological assessment; (4) a history of major neurological or psychiatric disorders, such as dementia, stroke, or depression. After screening, a total of 211 subjects (138 Non-MetS and 73 MetS patients) were enrolled in this study.

**Figure 1:**
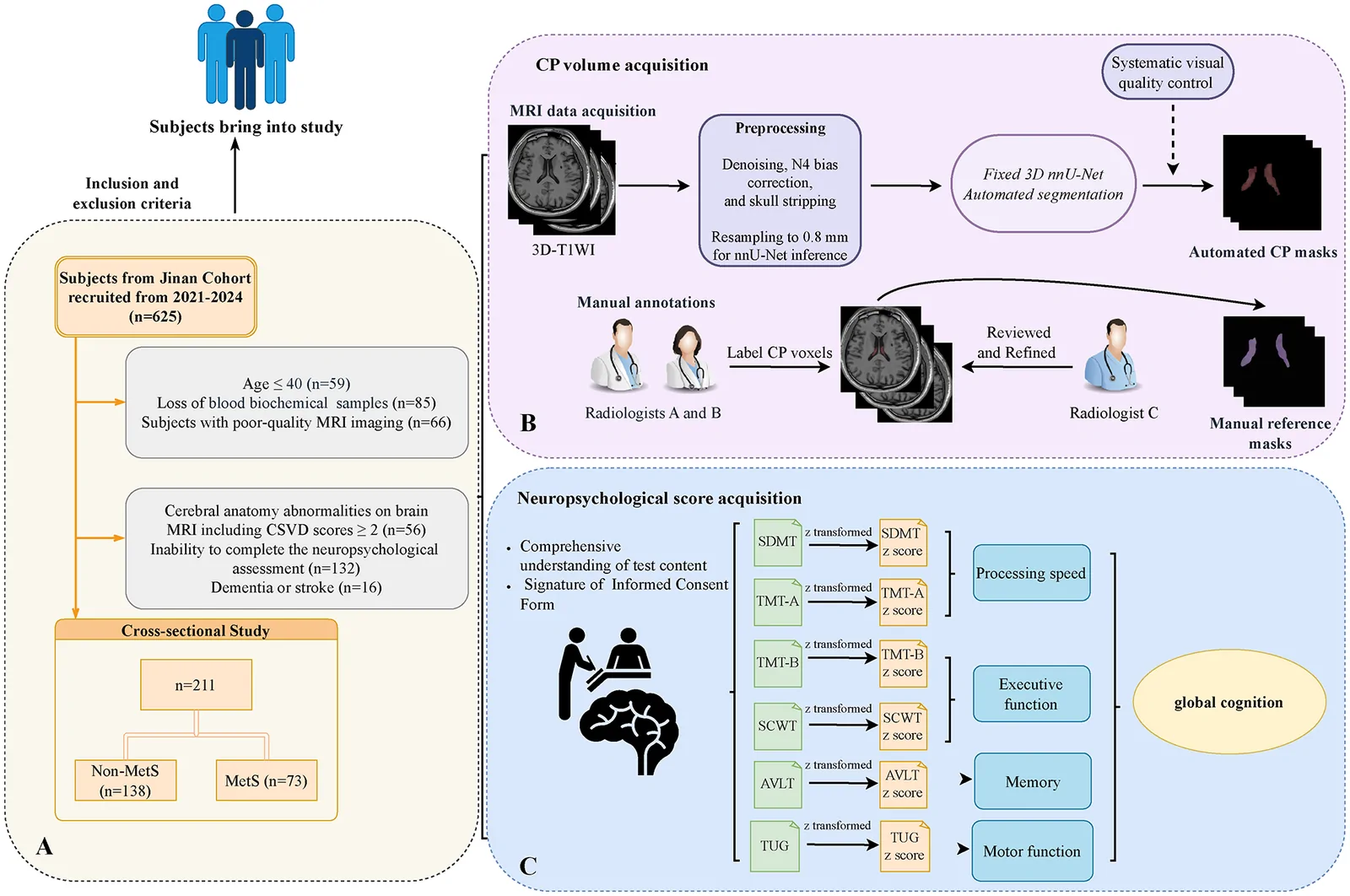
Participants of Jinan cohort selection process and inclusion/exclusion criteria and acquisition of CP volume and cognitive function. **(A)** Inclusion and exclusion criteria for subjects. **(B)** Segmentation flow chart of CP volume. **(C)** z-transformed process of cognitive functions. Cognitive test results were z-transformed separately and divided into four domains (processing speed, executive function, memory, motor function) and integrated them into global cognitive-motor composite. Processing speed~ TMT-A and SDMT; Executive function~TMT-B and SCWT; Memory~AVLT; Motor function~TUG. MetS, metabolic syndrome; 3D-T1W images, three-dimensional T1-weighted images; MRI, Magnetic Resonance Imaging; CP volume, choroid plexus volume; SCWT, Stroop Color and Word Test; TMT, Trail Making Test; AVLT, Auditory Verbal Learning Test; SDMT, Symbol Digit Modalities Test; TUG, timed up and go.

### 2.2 MRI acquisition

All the subjects underwent three-dimensional T1-weighted imaging (3D-T1WI) and diffusion tensor imaging (DTI) with a 3.0-T MR system (MAGNETOM Skyra, Siemens Healthcare, Erlangen, Germany) with a 32-channel head coil. 3D-T1W images were acquired via the following parameters: TR= 2,300 ms; TE= 2.32 ms; scan matrix = 256 × 256; FOV = 240 × 240 mm^2^; flip angle = 9°; and voxel size = 0.9 × 0.9 × 0.9 mm^3^. DTI images were obtained using a single-shot echo planar imaging sequence based on Diffusion-weighted imaging (DWI). The parameters are as follows: TR= 3,900 ms; TE= 65 ms; flip angle = 180°. In addition, T2-weighted turbo spin echo and T2-weighted fluid-attenuated inversion recovery were also acquired during scanning to ensure that the subjects had no brain lesions.

### 2.3 CP volume, WMH volume, and ALPS-index measurements

Figure 1B illustrates the CP segmentation and measurement workflow. CP masks were generated from 3D T1-weighted MRI scans using a fixed 3D nnU-Net segmentation pipeline previously developed by our group (24). The model was developed using a dataset independent of the present analytical cohort. None of the 211 participants included in the current study was used for model training, validation, testing, model selection, or parameter optimization.

For clinical analyses, CP volumes were derived from fully automated nnU-Net masks that passed visual quality control (Supplementary Table S1). All automated masks from the 211 analytical participants were reviewed by Dr. Li to assess anatomical plausibility, mask continuity, and obvious non-CP artifacts. To independently evaluate automated segmentation performance, we compared the nnU-Net outputs with manual reference masks in all 211 participants in the analytical cohort. The manual reference masks were primarily used as an independent reference standard for evaluating automated segmentation performance. The median Dice similarity coefficient was 0.983 (IQR: 0.918–0.992), and the median average surface distance was 0.064 mm (IQR: 0.019–0.135 mm), as reported in Supplementary Table S2. Detailed segmentation of CP volume, WMH volume, and the diffusivity along the perivascular space (ALPS) index measurements can be found in Appendix S1.

### 2.4 Clinical assessment

First, basic clinical data, including age, sex, years of education, medical history of hypertension and diabetes mellitus, and hyperlipidemia, smoking status, and alcohol consumption status, were collected. History of hyperlipidemia was defined as a documented clinical diagnosis of elevated blood lipids at enrollment. The total count of current medication classes consumed by each participant was documented. Blood biochemical examination and multiple comprehensive cognitive tests were subsequently performed on each subject. Multiple cognitive tests were performed in this research, including the Stroop Color and Word Test (SCWT) (25), Trail Making Test (TMT) including two components: TMT-A and TMT-B (26), Auditory Verbal Learning Test (AVLT) (27), Symbol Digit Modalities Test (SDMT) (28), and Timed Up and Go (TUG) (29).

Cognitive function was evaluated across four domains: processing speed (TMT-A and SDMT), executive function (TMT-B and SCWT), memory (AVLT), and motor function (TUG). We then integrated them into global cognitive-motor composite. Each test was z-transformed separately for participants (Tests for which higher values indicated poorer performance were reverse-scored.) and global cognitive-motor composite was defined as mean z score of all domains (30, 31). Details of the z-transformation procedure can be found in Figure 1C. All tests were conducted by professionals in a stationary and quiet venue.

### 2.5 Statistical analysis

The basic clinical characteristics of the participants and the results of multiple cognitive tests were collected. After the Shapiro-Wilk test was performed, we found that the measurements of CP volume, estimated total intracranial volume (eTIV) and their ratio were consistent with a normal distribution. Therefore, differences in categorical variables, such as sex, smoking status and drinking status, are expressed as percentages via the chi-square test. Two independent sample *t tests* and the Mann-Whitney U test were used for normally distributed continuous variables and skewed data, respectively, between participants with MetS and non-MetS. The most significant component of the CP volume changes was evaluated by multivariable linear regression after adjusting for age, sex, years of education, smoking, drinking and medication history by three models. The CP/eTIV ratio (choroid plexus volume divided by estimated total intracranial volume and multiplied by 10^4^) was used as the dependent variable, and the MetS components served as the independent variables. Using manual-reference CP masks, sensitivity analyses repeated in exploring the associations of CP/eTIV ratio with HDL-C and with MetS in the same statistical methods of the primary analyses. Spearman correlation analyses were employed to calculate the correlation coefficients between the CP/eTIV ratio, which were presented in previous research (32), MetS severity and cognitive-motor domains. Multivariable linear regression was used to examine the association between the CP/eTIV ratio and each cognitive-motor domain adjusted for age, sex, education, MetS status and lateral ventricular volume (LVV). *P* values in multivariate linear regression and correlation analyses were adjusted for multiple comparisons using the Benjamini-Hochberg false discovery rate (FDR) method.

Furthermore, the Boruta algorithm serves as an exploratory feature selection approach. The analysis was conducted on the complete dataset, with *p* Value = 0.05, maxRuns = 1,000, and a fixed random seed (set.seed (123)) to improve reproducibility. Tentative variables were resolved using the Tentative Rough Fix procedure. Boruta relies on a random forest framework for variable importance estimation. The random Forest package was used with default settings. Variables retained after Boruta screening were subsequently entered into a logistic regression model, and receiver operating characteristic (ROC) analysis was used to evaluate the ability of these predictors to distinguish participants with lower global cognitive-motor composite (below the 25% quantile of the global cognitive-motor composite) from the others. The 95% confidence interval of the area under the ROC curve (AUC) was estimated by bootstrap resampling with 2,000 iterations, and ROC curves were compared using DeLong's test. Neither cross-validation nor an independent test dataset was used, and feature selection was conducted on the full sample. The details of these models can be found in Appendix 1. The statistical threshold for all analyses was set at *P* < 0.05. All statistical analyses were performed using SPSS version 27.0 software (SPSS Inc., Chicago, IL, USA) and RStudio software (version 2023.06.2).

## 3 Results

### 3.1 Demographic and clinical characteristics

Table 1 shows the demographic details and clinical characteristics of each group at baseline. We found that there were no significant differences in age, LVV, eTIV, and multiple cognitive-motor domains between participants with MetS and non-MetS, but there were significant differences in sex, years of education, BMI, HbA1c, FPG, TG, HDL-C levels, ALPS-index and white matter hyperintensity (WMH) volume between the two groups. Additionally, the prevalence of smoking, alcohol consumption, hyperlipidemia, hypertension and diabetes mellitus in the MetS group was also significantly greater than that in the non-MetS.

**Table 1:** Baseline characteristics of the two groups.

Characteristic	MetS (*n* = 73)	Non-MetS (*n* = 138)	*χ^2^*/t/Z/U	*P* value
Age (years), mean (±SD)	59.6 (9.0)	58.4 (8.0)	−0.990	0.323^t^
Sex [male, *N* (%)]	53 (72.6)	59 (42.8)	17.080	< 0.001*^χ2^*
Education (years), median (IQR)	12 (12, 15)	15 (12, 16)	−2.254	0.024^a^
Smoking, [yes, *N* (%)]	31 (42.5)	22 (15.9)	17.857	< 0.001*^χ2^*
Alcohol consumption, [yes, *N* (%)]	40 (54.8)	38 (27.5)	15.225	< 0.001*^χ2^*
Number of current medication classes, median (IQR)	1 (0, 2)	0 (0, 1)	3169	< 0.001^a^
Metabolic characteristics				
History of hyperlipidaemia, [yes, *N* (%)]	58 (79.5)	53 (38.7)	31.761	< 0.001*^χ2^*
Hypertension, [yes, *N* (%)]	55 (75.3)	31 (22.5)	55.289	< 0.001*^χ2^*
Diabetes mellitus, [yes, *N* (%)]	59 (80.8)	40 (29.0)	51.551	< 0.001*^χ2^*
BMI (kg/m^2^), median (IQR)	26.84 (25.68, 28.40)	23.98 (22.16, 25.70)	−6.937	< 0.001^a^
HbA1c (%), median (IQR)	7.90 (6.80, 9.65)	5.90 (5.58, 6.80)	−6.086	< 0.001^a^
FPG (mmol/L), median (IQR)	7.86 (6.97, 10.55)	5.65 (5.09, 6.55)	−7.709	< 0.001^a^
TG (mmol/L), median (IQR)	1.96 (1.61, 2.37)	1.25 (0.89, 1.52)	−7.497	< 0.001^a^
HDL-C (mmol/L), median (IQR)	1.17 (1.17, 1.37)	–	–	< 0.001^a^
Neuropsychological assessments^*^				
Processing speed, median (IQR)	0.01 (−0.60, 0.60)	0.23 (−0.52, 0.79)	−1.077	0.281^a^
Executive function, median (IQR)	0.07 (−0.63, 0.48)	0.27 (−0.25, 0.64)	−1.92	0.055^a^
Memory, mean (±SD)	0.05 (1.05)	−0.03 (0.98)	−0.531	0.596^t^
Motor function, median (IQR)	−0.12 (−0.47, 0.36)	0.06 (−0.49, 0.66)	−1.615	0.106^a^
Global cognitive-motor composite, median (IQR)	0.07 (−0.55, 0.46)	0.09 (-0.32, 0.51)	−1.088	0.277^a^
Image features				
LVV, [×10^4^ mm^3^, median (IQR)]	2.08 (1.53,2.82)	1.83 (1.41, 2.49)	−1.797	0.072^a^
CP volume, [mm^3^, mean (±SD)]	1488.76 (460.70)	1246.72 (384.10)	−4.058	< 0.001^t^
ALPS-index, mean (±SD)	1.74 (0.199)	1.80 (0.24)	−2.116	0.036^t^
eTIV, [×10^6^ mm^3^, mean (±SD)]	1.50 (0.15)	1.47 (0.12)	−1.135	0.259^t^
WMH volume/eTIV, [×10^4^, median (IQR)]	7.56 (2.54, 21.56)	4.23 (2.08, 11.20)	5555	0.025^a^
CP volume/eTIV, [×10^4^, mean (±SD)]	10.0 (3.10)	8.0 (2.60)	−3.834	< 0.001^t^

Values are presented as mean (SD), median (IQR), or n (%), as indicated. ^χ2^ Chi-square test; ^t^ two independent sample t test; ^a^ Mann–Whitney U test. ^*^ Each neuropsychological test was z-transformed separately across all participants, and global cognitive-motor composite was defined as the mean z score across the four cognitive domains. MetS, metabolic syndrome; BMI, body mass index; HbA1c, glycosylated hemoglobin; FPG, fasting plasma glucose; TG, triglyceride; HDL-C, high-density lipoprotein cholesterol; LVV, lateral ventricle volume; WMH, white matter hyperintensity; CP, choroid plexus; ALPS, diffusivity along the perivascular space; eTIV, estimated total intracranial volume.

### 3.2 Choroid plexus volume

An obvious visual difference can be observed in Figure 2, which presents 3D T1-weighted images and comparison diagram of the CP volume between the two groups. Compared with non-MetS, participants with MetS presented a larger raw CP volume (*P* < 0.001) and a higher CP/eTIV ratio (*P* < 0.001). In the sensitivity analysis, manual-reference CP volume/eTIV was significantly higher in the MetS group than in the non-MetS group (*P* < 0.001) (Table S3).

**Figure 2:**
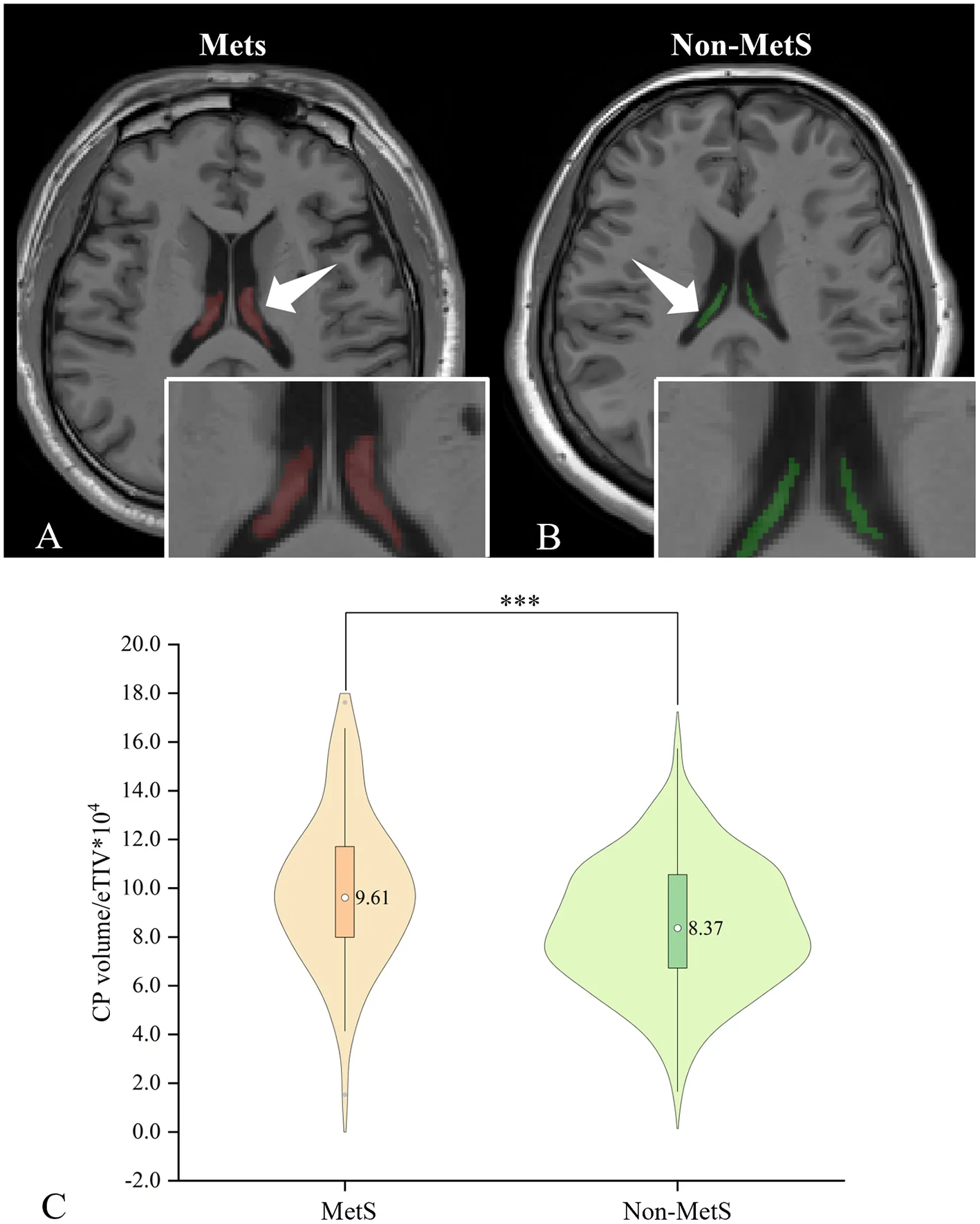
Comparison of the CP volume of the participants with MetS and non-MetS. **(A, B)** Representative images of the two groups showing 3D-T1-weighted images of the CP volume. The CP volume of participants with metabolic syndrome was greater than that of non-MetS. **(C)** Violin plots revealed a higher CP/eTIV ratio (*P* < 0.001) in the participants with MetS than in the non-MetS. Statistical results: * *P* < 0.05; ** *P* < 0.01; *** *P* < 0.001. MetS, metabolic syndrome; CP volume, choroid plexus volume; eTIV, estimated total intracranial volume.

### 3.3 Association of CP volume with MetS and its components

We explored the correlation of CP volume with clinical indicators and cognitive tests in all participants. Comparing the three models, we discovered that the MetS status was significantly correlated with a higher CP/eTIV ratio after full adjustment (Model 3: B = 0.974, 95% CI: 0.110 to 1.837, *P* = 0.027) (Table 2A). Among individual components, HDL-C was the only component that remained significantly associated with CP/eTIV ratio after correction for multiple comparisons, consistently showing an inverse effect (Model 3: B = −1.553, 95% CI: −2.587 to −0.520, *FDR q* = 0.024) (Table 2A). Although CP/eTIV increased with the number of MetS components (Model 1: B = 0.639, *P* < 0.001; Model 2: B = 0.291, *P* = 0.049), this effect was attenuated to borderline significance after full adjustment (Model 3: B = 0.318, *P* = 0.066) (Table 2B). Similarly, subjects with four MetS components had relatively higher CP/eTIV ratio compared to those without (Model 3: B = 1.827, 95% CI: 0.046 to 3.607, *P* = 0.045), but it was not significant after FDR correction (*FDR q* = 0.225) (Table 2B). The associations of the manual-reference CP/eTIV ratio with MetS and with HDL-C were evaluated using the same covariate-adjustment models as in the primary analysis (Table S4). In Model 3, the MetS group showed a significantly higher CP/eTIV ratio (B = 0.904, 95% CI: 0.060 to 1.747, *P* = 0.036), while HDL-C was inversely associated with the ratio (B = −1.672, 95% CI: −2.749 to −0.595, *P* = 0.003). The directions and magnitudes of these associations were consistent with those obtained from the automated segmentations.

**Table 2A:** Associations of MetS status and metabolic characteristics with the CP/eTIV ratio.

	Model 1			Model 2			Model 3		
Characteristic	B (95% CI)	*P* value	*FDR q*	B (95% CI)	*P* value	*FDR q*	B (95% CI)	*P* value	*FDR q*
Presence of MetS	1.709 (0.942, 2.475)	< 0.001	-	0.888 (0.109, 1.668)	0.026	-	0.974 (0.110, 1.837)	0.027	-
Metabolic characteristics									
Hypertension	0.990 (0.229, 1.750)	0.011	0.017	0.171 (−0.573, 0.915)	0.651	0.651	0.006 (−0.845, 0.856)	0.989	0.989
Diabetes mellitus	1.494 (0.757, 2.230)	< 0.001	< 0.001	0.562 (−0.195, 1.319)	0.145	0.418	0.562 (−0.297, 1.420)	0.198	0.446
History of hyperlipidaemia	1.061 (−0.398, 2.521)	0.153	0.153	0.847 (−0.500, 2.193)	0.216	0.418	0.790 (−0.623, 2.202)	0.271	0.446
HbA1c	0.296 (0.085, 0.507)	0.006	0.016	0.126 (−0.082, 0.335)	0.234	0.418	0.144 (−0.088, 0.375)	0.223	0.446
FPG	0.197 (0.042, 0.352)	0.013	0.017	0.066 (−0.089, 0.222)	0.400	0.457	0.077 (−0.107, 0.261)	0.407	0.465
BMI	0.131 (0.031, 0.231)	0.010	0.017	0.050 (−0.047, 0.147)	0.312	0.418	0.052 (−0.048, 0.152)	0.308	0.446
TG	0.291 (−0.056, 0.638)	0.10	0.114	0.168 (−0.160, 0.496)	0.314	0.418	0.170 (−0.176, 0.515)	0.335	0.446
HDL-C	−2.333 (−3.351, −1.316)	< 0.001	< 0.001	−1.529 (−2.530, −0.528)	0.003	0.024	−1.553 (−2.587, −0.520)	0.003	0.024

**Table 2B:** Associations of MetS severity (number of components) with the CP/eTIV ratio.

	Model 1			Model 2			Model 3		
Number of MetS components	B (95% CI)	*P* value	*FDR q*	B (95% CI)	*P* value	*FDR q*	B (95% CI)	*P* value	*FDR q*
Per component increment	0.639 (0.370, 0.907)	< 0.001	-	0.291 (0.001, 0.581)	0.049	-	0.318 (−0.021, 0.657)	0.066	-
0 (ref)	-	-	-	-	-	-	-	-	-
1	0.849 (−0.291, 1.988)	0.144	0.146	0.598 (−0.536, 1.733)	0.300	0.496	0.589 (−0.595, 1.773)	0.328	0.491
2	1.103 (0.029, 2.177)	0.044	0.073	0.070 (−1.005, 1.146)	0.898	0.898	0.061 (−1.147, 1.268)	0.921	0.921
3	2.239 (1.136, 3.343)	< 0.001	< 0.001	0.928 (−0.245, 2.100)	0.120	0.300	1.043 (−0.262, 2.347)	0.116	0.290
4	3.101 (1.600, 4.602)	< 0.001	< 0.001	1.699 (0.181, 3.216)	0.028	0.140	1.827 (0.046, 3.607)	0.045	0.225
5	1.685 (−0.594, 3.964)	0.146	0.146	1.001 (−1.328, 3.330)	0.397	0.496	1.057 (−1.380, 3.495)	0.393	0.491

Model 1: unadjusted. Model 2: adjusted for sex, age, and education. Model 3: adjusted for sex, age, education, smoking, drinking, and number of current medication classes. *P* values were adjusted for multiple comparisons within each model using the Benjamini-Hochberg false discovery rate (FDR) method. Adjusted *P* values (*FDR q*) are presented. The dependent variable was the CP/eTIV ratio, calculated as CP volume divided by eTIV and multiplied by 10^4^. B represents the unstandardized linear regression coefficient. CI, confidence interval; MetS, metabolic syndrome; BMI, body mass index; HbA1c, glycosylated hemoglobin; FPG, fasting blood glucose; TG, triglyceride; HDL-C, high-density lipoprotein cholesterol; eTIV, estimated total intracranial volume.

Unadjusted Spearman analyses showed that higher CP/eTIV ratio was correlated with poorer processing speed, executive function, motor function, and global cognitive-motor composite (Figure S1). However, these associations were no longer evident in multivariable linear regression after adjustment for age, sex, education, MetS status and LVV (all *P* > 0.05; Table S5).

### 3.4 Random forest and ROC curve analysis results

The importance ranking of variables associated with cognitive status, as assessed by the random forest model, is shown in Figure 3A. After 999 iterations in Boruta algorithm, three variables (CP/eTIV, WMH/eTIV, BMI) were identified as contributing important features (Figure S2). These three indicators were used to construct a combined model for discriminating participants below the 25th percentile of global cognitive-motor composite in the full sample. The apparent AUC of the combined model (CP/eTIV + WMH/eTIV + BMI) was 0.787 (95% CI: 0.718 to 0.854). Figure 3B displays the ROC curves for the individual and combined models. The AUCs for the individual indicators were 0.742 (95% CI: 0.668 to 0.818) for CP/eTIV, 0.689 (95% CI: 0.602 to 0.769) for WMH/eTIV, and 0.621 (95% CI: 0.540 to 0.701) for BMI. The model containing WMH/eTIV and BMI yielded an AUC of 0.729 (95% CI: 0.654 to 0.801). Adding CP/eTIV increased the AUC by 0.058 to 0.787 (Figure 3C). DeLong's test showed no significant differences of the AUC values between combined model and either CP/eTIV alone (0.742 vs. 0.787) or the model of WMH/eTIV and BMI (0.729 vs. 0.787). Significant differences were observed when comparing the combined model with WMH/eTIV alone (0.689 vs. 0.787, *FDR q* = 0.024) and BMI alone (0.621 vs. 0.787, *FDR q*=0.002) (Table 3).

**Figure 3:**
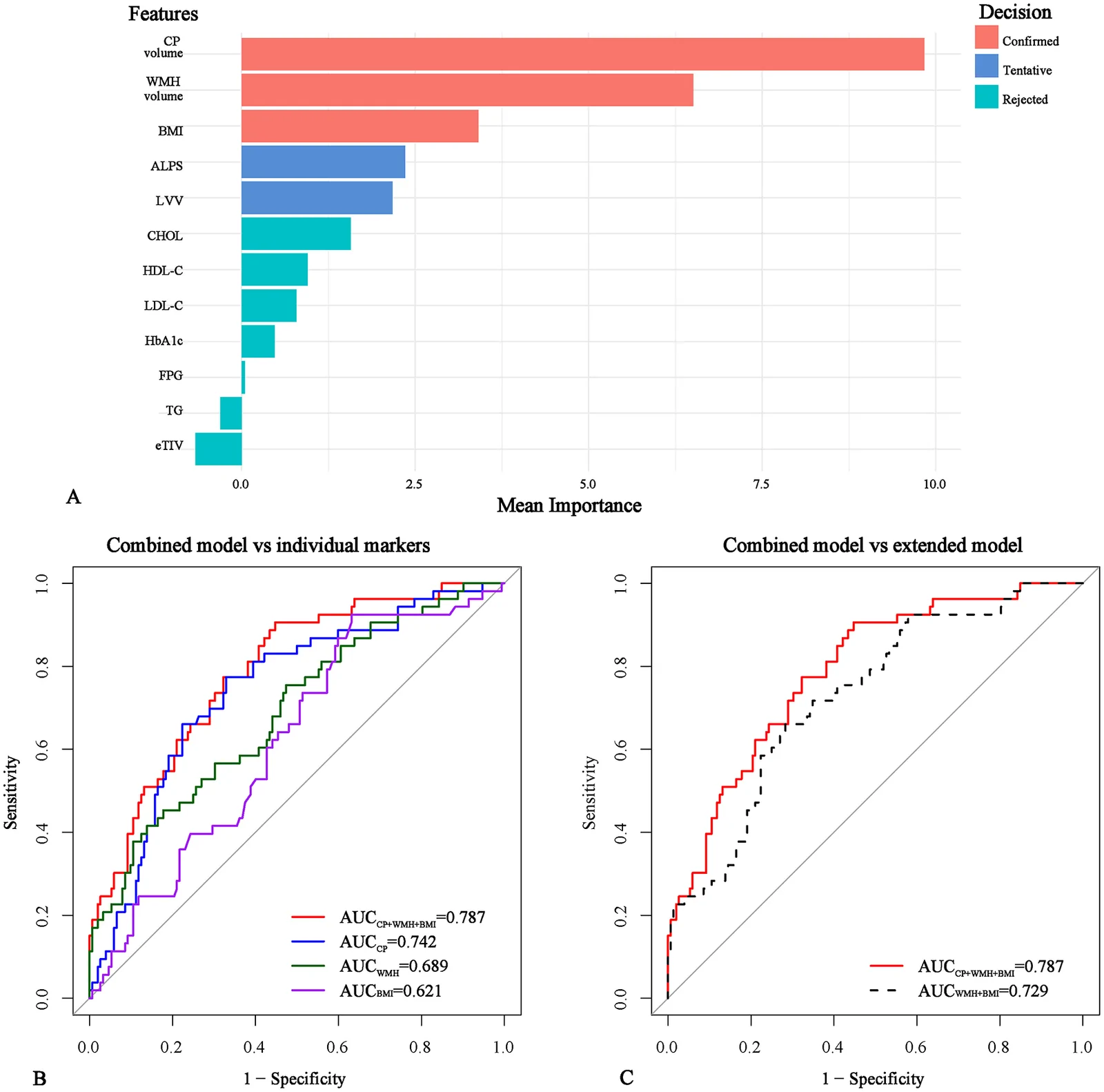
Exploratory feature selection and discrimination of lower global cognitive-motor performance. **(A)** demonstrates the feature importance rankings based on the Boruta algorithm, which are used to evaluate the contributions of various features in exploring the discrimination of lower global cognitive-motor performance. Features are categorized into three types: confirmed (red), rejected (green), and tentative (blue). The bar diagram represent the distribution of importance scores for each feature, with the x-axis indicating the feature importance and the y-axis listing the features. Confirmed features, such as CP/eTIV and WMH/eTIV show significantly greater importance. Rejected features have important scores that are lower than shadow features. **(B)** ROC curves for the model combining CP/eTIV, WMH/eTIV, and BMI, and single marker models. **(C)** ROC curves for the model combining CP/eTIV, WMH/eTIV, and BMI, and the model containing WMH/eTIV and BMI. CP/eTIV and WMH/eTIV represent CP volume and WMH volume, respectively, divided by eTIV and multiplied by 10^4^. ALPS, analysis along the perivascular space; AUC, area under the receiver operating characteristic curve; BMI, body mass index; CHOL, total cholesterol; CP, choroid plexus; eTIV, estimated total intracranial volume; FPG, fasting plasma glucose; HbA1c, glycosylated hemoglobin; HDL-C, high-density lipoprotein cholesterol; LDL-C, low-density lipoprotein cholesterol; LVV, lateral ventricular volume; TG, triglycerides; WMH, white matter hyperintensity.

**Table 3:** Exploratory ROC analyses of CP/eTIV ratio, WMH/eTIV ratio, BMI, and combined models for lower global cognitive-motor performance.

Factors	Probability cutoff	SEN	SPE	AUC	95% CI	DeLong's test (*vs* CP/eTIV + WMH/eTIV + BMI)		
						*Z*	*P* value	*FDR q*
CP/eTIV+ WMH/eTIV+ BMI	0.183	0.906	0.553	0.787	0.718–0.854	-	-	-
WMH/eTIV+BMI	0.238	0.660	0.717	0.729	0.654–0.801	1.783	0.075	0.084
CP/eTIV	0.250	0.774	0.671	0.742	0.668–0.818	1.729	0.084	0.084
WMH/eTIV	0.186	0.755	0.526	0.689	0.602–0.769	2.512	0.012	0.024
BMI	0.213	0.925	0.368	0.621	0.540–0.701	3.464	< 0.001	0.002

*P* values were adjusted across the four pairwise DeLong's tests with the combined model using the Benjamini-Hochberg false discovery rate (FDR) method. Adjusted *P* values (*FDR q*) are presented. Cutoffs are predicted probabilities from logistic regression models. CP/eTIV and WMH/eTIV represent CP volume and WMH volume, respectively, divided by eTIV and multiplied by 10^4^. ROC, receiver operating characteristic; AUC, area under the curve; SEN/SPE, sensitivity/specificity corresponding to maximum Youden index; CP volume, choroid plexus volume; WMH, white matter hyperintensity; BMI, body mass index; eTIV, estimated total intracranial volume.

## 4 Discussion

This study revealed that increased CP/eTIV in participants with MetS was significantly related to the decreased HDL-C after covariate adjustment. However, after adjusting for age, sex, education, MetS status, and LVV, CP/eTIV was not independently associated with any cognitive-motor domain. This research focuses on CP, a relatively underexplored structure in MetS. We utilized linear regression to explore metabolic risk factors on CP, random forest analysis identified CP/eTIV, WMH/eTIV, and BMI as key variables for discriminating participants with lower global cognitive-motor performance, and ROC analyses were used to evaluate their discriminative capacity in an exploratory framework. For CP segmentation, we employed 3D nnU-Net algorithm, a deep learning architecture that automates the segmentation process. Unlike traditional simpler machine learning algorithms, this method uses a multiscale approach that captures both fine and coarse features of the CP, significantly reducing human effort involvement and minimizing errors (33). The sensitivity analysis using manual-reference CP volumes yielded a consistent MetS-related group difference, supporting the robustness of the principal finding across segmentation approaches. Taken together, these exploratory findings suggest that CP volume is associated with certain metabolic components, particularly HDL-C, but its independent relationship with cognitive-motor performance and its incremental discriminative value remain to be established in larger prospective studies.

In our study, patients with MetS exhibited a higher CP/eTIV ratio than non-MetS controls, and this association remained significant even after adjustment for measured confounders. The possible explanations are as follows. Potential mechanisms underlying brain structural alterations in subjects with MetS include impaired vascular reactivity, neuroinflammation, oxidative stress, and abnormal lipid metabolism in the brain (7). A recent study demonstrated that the development and progression of MetS are interconnected with different inflammatory signaling pathways (34). The release of multiple proinflammatory cytokines is a result of increased visceral fat and adipocyte dysfunction during the pathophysiology of MetS (35). Moreover, local or systemic inflammatory cytokines can also promote the progression of inflammation in metabolic syndrome through inflammatory signaling pathways (18). Notably, the CP plays a key role in regulating the production of CSF, and the glymphatic system participates in the clearance of metabolic waste and bulk movement of CSF (13, 36). It has been proposed that increased CP volume may affect glymphatic function (35). In addition, previous studies have suggested that increased CP volume is associated with increased severity of central inflammation and decreased CSF clearance (37). Therefore, one possible explanation is that the increase in CP volume reflects a series of neuroinflammatory changes.

Reduced circulating HDL-C, one of the components of MetS, remained inversely associated with CP volume after adjusting for all included covariates. A previous study found that waist circumference was positively associated with CP volume and proposed obesity-related inflammation, diet, or abnormal leptin transport as possible explanations (22). CP enlargement has also been associated with peripheral inflammatory burden in specific disease cohorts (38). Peripheral lipoproteins do not freely cross the BBB, although they may influence the brain indirectly through cerebrovascular and brain-barrier interfaces (39–41). Taken together, these observations suggest that the association between HDL-C and CP volume may reflect broader systemic metabolic and inflammatory health rather than a direct effect of HDL on CP structure.

As previously mentioned, multiple inflammatory factors and inflammatory signaling pathways are associated with the occurrence and development of MetS, such as TNF-α and nuclear factor-κB (NF-κB) (34). Various immune cells and inflammatory cytokines produced by peripheral immunity are thought to increase the permeability of the BBB and then enter the CNS (42, 43). The impairment of the brain barrier and inflammatory changes in the CNS may be among the reasons for the alterations in CP volume. Meanwhile, peripheral insulin resistance (IR) and oxidative stress may promote central IR, which has been proposed to drive AD pathology via tau hyperphosphorylation, amyloid-β (Aβ) overproduction, mitochondrial dysfunction, and reactive oxygen species (ROS) generation (44). Accumulation of metabolic byproducts may overload clearance pathways, potentially leading to compensatory CP enlargement, given that the CP plays a key role in waste removal and CSF regulation. IR-induced hyperglycemia can promote ROS generation and oxidative stress, which in turn may exacerbate mitochondrial dysfunction and aggravate IR (45). Taken together, these findings suggest that peripheral metabolic abnormalities may be associated with CP structural alterations, potentially through inflammatory mechanisms. Although the association between cumulative metabolic severity and CP volume did not withstand the FDR correction, the pattern of increased CP volume among participants with four MetS risk factors needs further investigation in larger studies. The effect of multiple MetS risk factors is not simply additive. However, the present cross-sectional study cannot determine whether this pattern reflects additive, interactive, or threshold effects.

In unadjusted analyses, CP/eTIV was nominally associated with several cognitive-motor domains and MetS severity. However, after adjusting for age, sex, education, MetS status, and lateral ventricular volume, CP/eTIV was not independently associated with performance in any cognitive-motor domain. MetS and its risk factors are involved in the pathological changes associated with several neurodegenerative diseases, and increased CP volume has been linked to inflammation (46). Moreover, previous clinical studies have reported a negative association between CP volume and CP permeability. Findings from glymphatic system research further suggest that structural alterations of the CP may be linked to impaired CSF production and clearance, which could contribute to the accumulation of pathological proteins such as amyloid-β, a hallmark of AD (19, 47, 48). Additionally, the primary supplying arteries of the CP are the anterior choroidal artery, which usually arises from the internal carotid artery, and posterior choroidal branches of the posterior cerebral artery (49). Subjects with more severe MetS often have pathophysiological changes in cerebral arteriosclerosis (7), and cerebrovascular dysfunction is regarded as a contributor to both vascular dementia and AD (50). These cerebrovascular alterations may be associated with a decrease in cerebral perfusion and blood flow, which may in turn affect blood perfusion in the CP. It has been hypothesized that decreased perfusion may lead to compensatory dystrophic hyperplasia of the CP, which could further suggest that CP structural alterations are potentially linked to impaired CSF clearance and waste accumulation (51). Taken together, these findings suggest a possible role of the CP in cognitive-motor performance, although the proposed mechanisms remain to be directly validated in larger cohorts of MetS.

Recently, a study demonstrated that CP volume has clinical value in differentiating dementia, mild cognitive impairment, and normal cognition in the AD continuum (32). In our cohort, participants were generally over 40 years of age, and most of them had metabolic risk factors but had not yet developed overt MetS. The present findings raise the possibility that CP/eTIV might also help distinguish subjects with lower global cognitive-motor performance in populations with metabolic risk.

WMH, reflecting vascular injury and white matter disruption, has long been associated with both MetS and cognitive impairment (52). In exploratory ROC analyses, CP/eTIV emerged as a key feature in the random forest model. The combined model (CP/eTIV + WMH/eTIV + BMI) yielded an apparent AUC of 0.787, compared with 0.742 for CP/eTIV alone and 0.729 for the model of WMH/eTIV and BMI. However, DeLong's test indicated that the addition of CP/eTIV did not significantly improve discrimination beyond WMH/eTIV and BMI. Together, these results highlight the potentially complementary roles of CP and WMH, capturing putative neuroinflammatory and vascular injury pathways, respectively. Given the cross-sectional design and the absence of independent validation, these findings should be considered exploratory results.

Our research has numerous aspects to improve. First, there were differences in the sex ratio and education level between the two groups, and we used sex and education level as covariates to reduce this bias. Future studies should aim to recruit subjects matched for sex and education. Second, because this was a cross-sectional study, associations between CP volume, metabolic abnormalities, and cognitive-motor performance should not be interpreted as evidence of causality or prediction. We could not determine whether CP enlargement preceded metabolic dysfunction or cognitive decline, or whether these changes occurred at the same time. Longitudinal validations are needed to clarify these causal relationships and evaluate the significance of CP enlargement. Third, we defined subjects below the 25th percentile of global cognitive-motor composite as the low cognition group. Although this data-driven cutoff is widely adopted in epidemiological research to identify individuals with relatively lower global cognitive-motor performance, it has notable limitations. This cutoff is sample-dependent and may vary across different cohorts, limiting external generalizability. More importantly, it does not align with established clinical criteria for mild cognitive impairment or dementia. As such, the low cognition in our research reflects relative cognitive vulnerability within this specific cohort rather than a clinically diagnosed state. This limits the direct applicability of our findings to clinical populations, and future studies need to incorporate standardized clinical assessments to validate the observed associations in diagnostically confirmed populations. Fourth, although our regression models adjusted for key demographic and lifestyle factors, we lacked data on several potentially important confounders, including physical activity, comprehensive socioeconomic indicators, medication effects, and detailed vascular comorbidities. Fifth, CP is a small and anatomically variable structure, partial-volume effects and anatomical variability may therefore influence volume estimates. Resampling standardized model input but did not eliminate the spatial-resolution limitations of the native MRI data, and external validation using harmonized or higher-resolution imaging is warranted. Feature selection and model evaluation in this study serve as exploratory results. Future studies with richer phenotypic characterization and independent external validation cohorts are warranted to validate and extend our findings. We hope that combination of cross-sectional design and longitudinal studies will be valuable to capture dynamic cognitive changes over time in the future.

## 5 Conclusions

These findings suggest that higher CP/eTIV ratio is associated with MetS and lower circulating HDL-C levels and may represent a neuroimaging marker correlated with metabolic disturbance. However, CP/eTIV ratio was not independently associated with cognitive-motor performance after multivariable adjustment, and this relationship requires further evaluation in larger prospective studies.

## Data Availability

The raw data supporting the conclusions of this article will be made available by the authors, without undue reservation.
