# Supplementary material for: Choroid plexus volume in metabolic syndrome: a cross-sectional neuroimaging study
Source: Front Neurol. 2026 Oct 16;17:1912049. doi: 10.3389/fneur.2026.1912049 (PMC13721072; doi:10.3389/fneur.2026.1912049)
Supplement: Supplementary file 1 [file Supplementary_file_1.docx]

***Supplementary Materials***

**Appendix S1**

**1. CP volume collection and measurements**

**1.1 Image preprocessing**

All raw DICOM images were converted to NIfTI format and reoriented to the RAS convention. The preprocessing pipeline consisted of three main steps: denoising, N4 bias-field correction, and skull stripping implemented through a deep learning-based brain extraction algorithm. For segmentation employing the nnU-Net, images were uniformly resampled to achieve 0.8 mm isotropic resolution and subsequently normalized using z-scores in accordance with the 3D full-resolution configuration. The resulting segmentation masks were then transformed back to each participant's native 0.9 mm space for segmentation evaluation and volume quantification. Estimated total intracranial volume (eTIV) was derived using FreeSurfer (version 6.0.1).

**1.2 Manual delineation procedure for validation**

To establish manual reference masks for independent evaluation of automated segmentation, two senior radiologists (each with over 20 years of experience in central nervous system imaging) independently performed initial manual delineations of the CP using ITK-SNAP software (version 4.0). Regions presenting ambiguous boundaries, such as cerebrospinal fluid adjacent to the choroidal fissure, vascular structures, or calcifications, were conservatively omitted. Notably, choroid plexus tissue located in the third and fourth ventricles was excluded from the annotation protocol(1).

All initial manual segmentations were subsequently reviewed and refined through visual inspection by a third senior radiologist with 25 years of imaging diagnosis experience, who ensured anatomical accuracy and consistency across all cases. The final operator-verified masks were used as the manual reference standard for independent segmentation evaluation. In addition, manual-reference CP volumes derived from these final masks were normalized to eTIV and used in a limited sensitivity analysis of the associations of MetS status and HDL-C with CP volume. The primary clinical analyses remained based on automated CP volumes.

**1.3 Model development and training protocol**

A fixed single-label segmentation model for choroid plexus identification based on the nnU-Net framework (employing 3D full-resolution configuration with PlainConvUNet as the backbone architecture) was applied in the present study. The model-development dataset was independent of the present analytical cohort. None of the 211 participants included in this study was used for model training, validation, testing, model selection, or parameter optimization. Detailed model configuration parameters are provided in Table S1.

**1.4 Validation of automated segmentation accuracy**

To independently evaluate automated segmentation performance, we compared the initial nnU-Net outputs with the final manual reference masks in all 211 participants included in the analytical cohort. None of these participants was included in model development or parameter optimization. The following metrics were computed in the native 0.9 mm isotropic space presented in **Table S2**: Dice similarity coefficient (DSC), Jaccard index, precision, recall, relative volume difference (%) and average surface distance (ASD, mm). The median DSC was 0.983 (IQR: 0.918–0.992), indicating high spatial overlap. The median ASD was 0.064 mm (IQR: 0.019–0.135 mm). The high precision (median 0.998) and moderate recall (median 0.972) indicate that the segmentation was conservative, with minimal false positive voxels. These metrics quantify the agreement between automated masks and independent manual reference masks in the present analytical cohort. All primary clinical statistical analyses used CP volume derived from automated nnU-Net masks that passed visual quality control. Manual reference masks were used for independent segmentation evaluation and for a sensitivity analysis using manual-reference CP volume.

**1.5 White matter hyperintensity volume**

Structural MRI data were preprocessed using fMRIPrep 20.1.1, which produced a nonlinear deformation field from native space to the MNI2009c space(2). WMH segmentation was carried out with an optimized algorithm based on Multidimensional Gated Recurrent Units, integrating data augmentation, selective sampling, residual learning, and DropConnect in the RNN state(3). The algorithm used T1 W and FLAIR images to generate WMH masks in native T1 W space. WMH volumes were then extracted using fslstats.

**1.6 DTI‑ALPS postprocessing and ALPS calculation**

Diffusion tensor imaging (DTI) data were processed with FSL (version 5.0.9). The ALPS index was then calculated following previous studies(4). Briefly, diffusivity along perivascular spaces (PVSs) was referenced from the projection and association fibers at the lateral ventricle body level, identified as the superior corona radiata (SCR) and superior longitudinal fasciculus (SLF) using the JHU-ICBM-DTI-81 atlas(5). Diffusivity values (Dxx, Dyy, Dzz) were extracted from 5-mm spherical ROIs placed in bilateral SCR and SLF (MNI coordinates: left SCR 116,110,99; left SLF 128,110,99; right SCR 64,110,99; right SLF 51,110,99). The ALPS index was calculated as the ratio of mean x-axis diffusivity in projection and association fibers to the mean of y-axis diffusivity in projection fibers and z-axis diffusivity in association fibers.

**2. Statistical analysis**

**2.1 Details of Boruta-based feature selection**

To identify candidate variables associated with low cognitive status, we used the Boruta algorithm as an exploratory feature selection method in R. Analyses were conducted on the complete dataset. Boruta was implemented using the Boruta package (version 8.0.0) with pValue = 0.05, maxRuns = 1000, and a fixed random seed (set.seed(123)) to improve reproducibility. Tentative features were further resolved using the TentativeRoughFix procedure.

Because Boruta relies on a random forest framework for variable importance estimation, the randomForest package (version 4.7.1.2) was used with default settings. For the present classification analysis, these defaults were ntree = 500, mtry = floor(sqrt(p)), nodesize = 1, and maxnodes = NULL. This step was intended for exploratory variable screening rather than for development of a fully validated predictive model.

**2.2 ROC model construction**

Variables retained after Boruta screening were subsequently entered into a multivariable logistic regression model to distinguish participants with lower global cognitive-motor performance, defined as a global cognitive-motor score below the 25% quantile of the global cognitive-motor composite. Model discrimination was evaluated using ROC analysis with the pROC package (version 1.18.5). The area under the ROC curve (AUC), sensitivity, and specificity were calculated, and the 95% confidence interval of the AUC was estimated by bootstrap resampling with 2000 iterations. ROC curves were compared using DeLong’s test. No cross-validation was performed, no independent test dataset was used, and feature selection was not embedded in a nested validation framework.

Table S1. Configuration of the fixed nnU-Net pipeline for choroid plexus segmentation.

| **Category** | **Description** |
| --- | --- |
| Pipeline use | Fixed 3D nnU-Net pipeline; none of the 211 analytical participants was used for model development or optimization. |
| Input preprocessing | Resampling to 0.8-mm isotropic resolution and per-image z-score normalization. |
| Network architecture | nnU-Net 3D full-resolution configuration with a PlainConvUNet backbone. |
| Inference | Sliding-window prediction with mirrored test-time augmentation. |
| Output space | Predicted masks were transformed back to native 0.9-mm isotropic space before evaluation and volume quantification. |

Table S2. Agreement between automated and manual choroid plexus segmentations in the analytical cohort (n = 211).

| **Metric** | **Mean** | **SD** | **Median** | **Q1** | **Q3** |
| --- | --- | --- | --- | --- | --- |
| Dice | 0.934 | 0.094 | 0.983 | 0.918 | 0.992 |
| Jaccard | 0.889 | 0.145 | 0.967 | 0.849 | 0.985 |
| Precision | 0.961 | 0.091 | 0.998 | 0.991 | 1.000 |
| Recall | 0.911 | 0.106 | 0.972 | 0.852 | 0.990 |
| Relative volume difference (%) | 6.265 | 7.356 | 1.907 | 0.821 | 11.559 |
| HD95 (mm) | 0.877 | 3.660 | 0.000 | 0.000 | 0.938 |
| ASD (mm) | 0.258 | 0.552 | 0.064 | 0.019 | 0.135 |

Table S3. Comparison of manual-reference CP/eTIV between participants with MetS and non-MetS controls.

|  | **MetS** | **non-MetS** | **t** | ***P* value** |
| --- | --- | --- | --- | --- |
| CP/eTIV × 10⁴ (Mean ± SD) | 10.93 (3.21) | 9.37 (2.64) | -3.769 | <0.001^t^ |

Table S4. Sensitivity analyses of the associations of MetS status and HDL-C with manual-reference CP/eTIV.

| **Exposure** | **Model** | **B** | **95%CI_Lower** | **95%CI_Upper** | **t** | ***P* value** |
| --- | --- | --- | --- | --- | --- | --- |
| MetS | Model 1 | 1.557 | 0.742 | 2.371 | 3.769 | <0.001 |
| MetS | Model 2 | 0.954 | 0.169 | 1.740 | 2.395 | 0.018 |
| MetS | Model 3 | 0.904 | 0.060 | 1.747 | 2.112 | 0.036 |
| HDL-C | Model 1 | -2.359 | -3.438 | -1.280 | -4.312 | <0.001 |
| HDL-C | Model 2 | -1.716 | -2.772 | -0.661 | -3.207 | 0.002 |
| HDL-C | Model 3 | -1.672 | -2.749 | -0.595 | -3.061 | 0.003 |

Note: Model 1: unadjusted. Model 2: adjusted for sex, age, and education. Model 3: adjusted for sex, age, education, smoking, drinking, and number of current medication classes. The dependent variable was the Manual-reference CP/eTIV ratio, calculated as CP volume divided by eTIV and multiplied by 10^4^. B represents the unstandardized linear regression coefficient.

Abbreviations: CI, confidence interval; MetS, metabolic syndrome; BMI, body mass index; HbA1c, glycosylated hemoglobin; FPG, fasting blood glucose; TG, triglyceride; HDL-C, high-density lipoprotein cholesterol; eTIV, estimated total intracranial volume.

Table S5. Multiple linear regression of choroid plexus volume and cognitive performance.

| **Cognitive outcome** | **Model 1** | | | **Model 2** | | |
| --- | --- | --- | --- | --- | --- | --- |
|  | **B(95% CI)** | ***P*** | ***FDR q*** | **B(95% CI)** | ***P*** | ***FDR q*** |
| Processing speed | 0.002 (-0.084, 0.089) | 0.960 | 0.960 | 0.002 (-0.087, 0.092) | 0.959 | 0.965 |
| Executive function | 0.044 (-0.058, 0.146) | 0.394 | 0.657 | 0.042 (-0.065, 0.148) | 0.438 | 0.965 |
| Memory | 0.080 (-0.059, 0.220) | 0.258 | 0.644 | 0.039 (-0.104, 0.183) | 0.588 | 0.965 |
| Motor function | -0.111 (-0.262, 0.039) | 0.145 | 0.644 | -0.090 (-0.245, 0.065) | 0.253 | 0.965 |
| Global cognitive-motor composite | 0.004 (-0.066, 0.073) | 0.914 | 0.960 | -0.002 (-0.074, 0.071) | 0.965 | 0.965 |

Note. Model 1 was adjusted for age, sex, and years of education. Model 2 was additionally adjusted for metabolic syndrome (MetS) status and lateral ventricular volume (LVV). The variable was the CP/eTIV ratio (CP volume/eTIV×10^4^). *FDR q* values were calculated across the five cognitive outcomes within each model using the Benjamini-Hochberg procedure. B represents the unstandardized linear regression coefficient.

Abbreviations: MetS, metabolic syndrome; CP volume, choroid plexus volume.

**Figure S1. Heatmap showing the correlation of CP volume (choroid plexus volume adjusted for estimated total intracranial volume) and the severity of metabolic syndrome with multiple cognitive domains.** For the correlation matrix, all 21 pairwise correlations were tested, and *P* values were adjusted for multiple comparisons using the Benjamini-Hochberg false discovery rate (FDR) method. **Red** indicates a positive correlation and **Blue** indicates a negative correlation.

Statistical results: **FDR q* < 0.05; ***FDR q* < 0.01; ****FDR q* < 0.001.

Abbreviations: MetS, metabolic syndrome; CP volume, choroid plexus volume.

**Figure S2. Feature-selection frequency plot for exploratory discrimination of lower global cognitive-motor performance.**

Boruta algorithm performed 999 iterations and compared the importance of the original variables with the highest feature importance of the shadow features obtained via feature permuted copies. Shadow features serve as a baseline to assess the relevance of actual features. After selection, three variables were confirmed as important (CP/eTIV, WMH/eTIV, and BMI); two were tentative (ALPS-index and LVV); and seven were unimportant. CP/eTIV and WMH/eTIV represent CP volume and WMH volume, respectively, divided by eTIV and multiplied by 10^4^.

Abbreviations: BMI, body mass index; HbA1c, glycosylated hemoglobin; FPG, fasting blood glucose; TG, triglyceride; HDL-C, high-density lipoprotein cholesterol; LVV, lateral ventricle volume; WMH, White matter hyperintensity; CP volume, choroid plexus volume; ALPS, diffusivity along the perivascular space; eTIV, estimated total intracranial volume.

1.	Bannai D, Cao Y, Keshavan M, Reuter M, Lizano P. Manual Segmentation of the Human Choroid Plexus Using Brain MRI. Journal of visualized experiments : JoVE. 2023(202). Epub 2024/01/02. doi: 10.3791/65341. PubMed PMID: 38163279.

2.	Esteban O, Markiewicz CJ, Blair RW, Moodie CA, Isik AI, Erramuzpe A, et al. fMRIPrep: a robust preprocessing pipeline for functional MRI. Nature methods. 2019;16(1):111-6. Epub 2018/12/12. doi: 10.1038/s41592-018-0235-4. PubMed PMID: 30532080; PubMed Central PMCID: PMCPMC6319393.

3.	Müller J, Sinnecker T, Wendebourg MJ, Schläger R, Kuhle J, Schädelin S, et al. Choroid Plexus Volume in Multiple Sclerosis vs Neuromyelitis Optica Spectrum Disorder: A Retrospective, Cross-sectional Analysis. Neurology(R) neuroimmunology & neuroinflammation. 2022;9(3). Epub 2022/02/27. doi: 10.1212/nxi.0000000000001147. PubMed PMID: 35217580; PubMed Central PMCID: PMCPMC8883575.

4.	Liu X, Barisano G, Shao X, Jann K, Ringman JM, Lu H, et al. Cross-Vendor Test-Retest Validation of Diffusion Tensor Image Analysis along the Perivascular Space (DTI-ALPS) for Evaluating Glymphatic System Function. Aging and disease. 2024;15(4):1885-98. Epub 2023/06/13. doi: 10.14336/ad.2023.0321-2. PubMed PMID: 37307817; PubMed Central PMCID: PMCPMC11272201.

5.	Taoka T, Masutani Y, Kawai H, Nakane T, Matsuoka K, Yasuno F, et al. Evaluation of glymphatic system activity with the diffusion MR technique: diffusion tensor image analysis along the perivascular space (DTI-ALPS) in Alzheimer's disease cases. Japanese journal of radiology. 2017;35(4):172-8. Epub 2017/02/16. doi: 10.1007/s11604-017-0617-z. PubMed PMID: 28197821.
